# Designing Acceptable Robots for Assisting Older Adults: A Pilot Study on the Willingness to Interact

**DOI:** 10.3390/ijerph182010686

**Published:** 2021-10-12

**Authors:** Roberta Bevilacqua, Elisa Felici, Filippo Cavallo, Giulio Amabili, Elvira Maranesi

**Affiliations:** 1 IRCCS INRCA Scientific Direction, 60124 Ancona, Italy; r.bevilacqua@inrca.it (R.B.); e.felici@inrca.it (E.F.); e.maranesi@inrca.it (E.M.); 2 Dipartimento Ingegneria Industriale, Università degli Studi di Firenze, 50139 Firenze, Italy; filippo.cavallo@unifi.it; 3 Istituto di BioRobotica, Scuola Superiore Sant’Anna, 56025 Pontedera, Italy

**Keywords:** human-robot interaction, older people, technology acceptance, human-centered design, qualitative study, emotional design

## Abstract

The aim of this paper was to explore the psychosocial determinants that lead to acceptability and willingness to interact with a service robot, starting with an analysis of older users’ behaviors toward the Robot-Era platform, in order to provide strategies for the promotion of social assistive robotics. A mixed-method approach was used to collect information on acceptability, usability, and human–robot interaction, by analyzing nonverbal behaviors, emotional expressions, and verbal communication. The study involved 35 older adults. Twenty-two were women and thirteen were men, aged 73.8 (±6) years old. Video interaction analysis was conducted to capture the users’ gestures, statements, and expressions. A coded scheme was designed on the basis of the literature in the field. Percentages of time and frequency of the selected events are reported. The statements of the users were collected and analyzed. The results of the behavioral analysis reveal a largely positive attitude, inferred from nonverbal clues and nonverbal emotional expressions. The results highlight the need to provide robotic solutions that respect the tasks they offer to the users It is necessary to give older consumers dedicated training in technological literacy to guarantee proper, long-lasting, and successful use.

## 1. Introduction

The field of social robotics is an established and growing scientific area for applications in ageing that is intended to improve quality of life and the independent living of older people. The interaction between humans and robots has become particularly relevant, especially in the fields of gerontology and geriatrics, to the extent of developing several robots to provide companionship to older adults [1] and to support them in managing their health and lifestyle [1,2,3,4]. The benefits of these solutions can be ascribed to the regaining of autonomy and ensuring the quality of life of the final users, in terms of partially restoring independence and autonomy, reducing workload for caregivers, and economic savings for individuals and government. Within the most important solutions developed to date, Pearl (by the University of Pittsburgh and Carnegie Mellon University, Pittsburgh, PA, USA), Caro-o-bot (by Fraunhofer Institute for Manufacturing Engineering and Automation, Stuttgart, Germany), and RI-MAN (by Riken’s Bio-Mimetic Control Research Center) represent some good examples of assistive robots, as well as CareBot™ MSR 3.4, produced by Gecko Systems (Petersfield, United Kingdom). Moreover, in the last years, several projects have been funded in order to reach a good level of cognitive and sensory-motor skills of robotic solutions, such as navigation and learning (Robots@Home, Movement, Radhar, Europa, Mow-by-Sat, and Iuro projects), manipulation (Handle, and First- Mobile Manipulation (MM) projects), safe human–robot interaction and cognition (Chris, and Aliz-E projects), networks of robots and middleware (DustBot and Urus project), humanoid (Humavips project, Agile Co-Creation for Robots and Aging (ACCRA) project), assistance robots for older people (Culture-Aware Robots and Environmental Sensor Systems for Elderly Support (CARESSES) project), and learning and intelligence (Explorers project). Another recent project aiming at improving well-being in older adults in Europe and Japan, and thereby promoting active and healthy ageing, contributing to independent living, and reducing the risks of social exclusion in older adults, is the EU-Japan Virtual coach for smart ageing (e-VITA project, www.e-vita.coach accessed on 8 October 2021). In this trial, off-the-shelf robotic platforms, such as NAO and PEPPER, will be used to deliver coaching contents to older people.

From the definition of social assistive robotics (SARs), proposed by Mataric et al. [5], it is possible to recognize the ability to cooperate with users and social interaction as essential features of any robotic applications designed for the older population.

Several studies [6,7,8,9] found that the willingness to use and interact with technology depends on the end users’ perceptions about ease of use, usefulness, value, and impact on quality of life (QoL). Moreover, self-efficacy was found to be a strong predictor of long-term technology adoption [7,9,10], as well as having past experience with devices, and the availability of digital literacy training [7,11,12]. In particular, training is often desired by older adults [11], and this is relevant for building up their confidence with and trust in the device [9]. Additionally, factors predicting willingness to adopt a technology vary across types of devices [7,8]. Regarding robotics, older users tend to prefer healthcare robots that perform a wide range of activities, including housekeeping, manipulation of objects, and information management [13,14]. Indeed, older adults consider social robots useful for personal care, leisure activities, Instrumental Activities of Daily Living (IADLs), and Electronic Aids to Daily Living (EADLs) over Activities of Daily Living (ADLs) [12,13,15,16].

Despite this evidence, the unavailability of resources for technical development and the requirements imposed by the scarcity of flexible usage settings, such as in the private homes of potential users, still represent barriers for developers, who are often forced to prioritize the improvement in specific technical features, to the detriment of the social and communication capabilities of the robots [17].

On the assistance side, designing new psychosocial interventions to support the behavioral and psychological symptoms of dementia, promoting advanced care planning, improving nutrition and quality of life, supporting the maintenance of physical and social functions, designing lifestyle interventions, and supporting frailty [18] are only a few of the problems that can be solved through SARs. The purpose of developing solutions to perform these complex tasks may decrease the technical efforts devoted to improving the interpersonal capabilities of the robot, with the consequence that the most compelling request of older adults is disregarded: that of receiving personalized and human care [19]. In addition, since the greatest benefit of SARs is that older adults are given the opportunity to remain at home, it is fundamental to understand how to shape the relationship between user and robot, in order to favor interaction in the close “familiar” context.

The aim of this study was to explore the psychosocial determinants that lead to the acceptability and willingness to interact with SARs, with the aim of providing strategies to support older people/robot interactions, starting with an analysis of the nonverbal behavior, emotional expression, and verbal communication of thirty-five older adults involved in the validation of the Robot-Era platform.

In our case, the platform was able to cooperate with the users in numerous daily activities in an autonomous and safe way; the communication capability of the robot was related to the task execution and can be defined as “instrumental”. A mixed-method approach was used, which combined video observation of human-robot interaction (HRI) cues and content analysis of the verbal expressions of the users. The size of the sample represents one of the most relevant studies in terms of end-user involvement to the best of our knowledge.

The Robot-Era architecture consists of three robotic platforms that are able to work in different environments, including an ambient intelligence infrastructure [20]. The robotic agents involved in this system are the domestic robot (DORO), which operates at home; condominium robot, which works in the common areas of buildings (CORO); and outdoor robot (ORO), which moves in the streets.

DORO is the robot that directly interacts with the users. It is equipped with a robotic arm to pick up and carry small objects. Multicolor LEDs, mounted on the eyes, and speakers provide feedback to the user. The robot has a removable tablet that user can use for service requests. DORO was designed using the SCITOS G5 mobile platform, (Ilmenau, Germany, http://www.metralabs.com, accessed on 8 October 2021). A front laser (SICK S300, Düsseldorf, Germany) and a rear laser (Hokuyo URG-04LX, Hokuyo Automatic, Osaka, Japan), for a 360° field of vision, allow them to safely navigate and avoid obstacles. DORO is equipped with a Kinova Jaco arm for manipulation tasks and a removable tablet for service requests. The full platform description is reported in Cavallo et al. [21]. On the basis of end-users’ needs analysis conducted during the project, DORO was able to perform services such as shopping delivery, garbage collection, and communication support.

## 2. Materials and Methods

A pilot study was performed inside the DomoCasa Lab in Italy. A mixed-method approach was designed, combining qualitative and quantitative techniques to collect information about the acceptability, usability, and human–robot interaction (HRI) requirements to be improved.

The inclusion criteria for selection were: (1) age ≥ 65 years old; (2) subjects lived alone or with relatives, but without a devoted caregiver; (3) voluntary participation. Additional information is available in [22].

The participants were recruited through telephone, advertisements, announcements, and social media channels of local associations and municipalities. The final sample was composed of 35 users: 22 women and 13 men. All of them lived either independently (7), or with their partner (28). Their mean age was 73.8 (±6) years old.

After recruitment, the first step of the procedure was to welcome the participants to the Robot-Era project. A written informed consent form was signed by all of the participants before partaking in the experiment. Then, a video was shown, which explained the Robot-Era services and functionality. As a second step, three interaction sessions were conducted, one for each service. During the session, the users were free to test as they liked. The researcher gave instructions to the participants to perform the tasks, which gave them the opportunity of trying them out, and participants expressed their opinion during use. The participants were free to ask questions and discuss with the experimenters during the sessions. Each session was composed of tasks to be performed with DORO:

- Shopping interaction session: The participant had to create and send a shopping list using the Graphical User Interface (GUI) on DORO and wait for the shopping delivery. The other Robot-Era platforms were involved. ORO was supposed to move to the shopping center to pick up the goods that were requested by DORO through the cloud, and then carry them to CORO through a roller mechanism for exchange, that, after receiving them, was enabled to deliver them to the door of the apartment.
- Communication interaction session: DORO was supposed to interact with participants to alert about possible dangerous situations using GUI and speech; furthermore, DORO was also enabled by the participant through the GUI to establish remote audio/video teleconference to enable remote communication with caregivers.
- Garbage interaction session: The participant had to activate the garbage service using the GUI on DORO, selecting the garbage typology. Then, CORO was enabled to move in front of the apartment door and receive the garbage box from the participant; next, CORO was programmed to move in front of ORO and exchange garbage, using the roller mechanism, for further outdoor transportation to the garbage collection center.

During each session, video interaction analysis (VIA) was conducted to allow capturing of all the user’s gestures, statements, and expressions, in a reliable and permanent way. The videos were taken from three different perspectives: in front of the subject, in a lateral position for analyzing interactions with the robot, and behind the subject’s shoulders, to collect information about their interaction with the tablet. All video analysis sessions were conducted individually by three psychologists trained on the contents of the study and on how to interpret the end-user’s behavior and emotional reactions to the robot. The results represent the final consensus reached by the researchers [23,24].

### 2.1. Acceptability and Human–Robot Interaction Assessment

In order to study acceptability and human–robot interaction, it was decided to combine analyses of both nonverbal communication, and of the free statements provided by the users. In human–robot interaction (HRI) studies, it has become standard practice to use video coding in order to study how a robot’s behavior influences interaction [25,26]. This type of analysis can produce both qualitative data about the characteristics of interaction and quantitative data about the frequency of significant behaviors.

The first step was to build a set of indicators for collecting HRI clues, following the categories of Andrés et al. [27] regarding the analysis of end-users’ behavior, in particular:

- Emotion: enjoyment, anxiety, neutral;
- Proxemics: intimate, personal and social, public;
- Gaze: directed gaze, none;
- Verbal interaction with the robot: conceptual use, conversational interaction, no interaction.

During the video analysis, a number of nonverbal behaviors that were significant for the quality of interaction were identified to generate a coding scheme for interpreting the interactions. In particular, we collected nonverbal behaviors that indicated engagement of the users with the robot, such as gaze and body direction, that represented a way of investigating joint attention between the human and the robot. Behaviors indicating joint attention were shown to improve both task performance and people’s impressions of a robot [28]. In addition to these spatial behaviors, non-verbal, emotional behaviors by the user were recorded, i.e., smiling.

Due to the specific experimental protocol that allowed a conceptual use of the robot, our hypothesis was that the duration of these tasks depended directly on the users’ willingness to interact. In this regard, the length of the interaction of each task, computed in percentage, could be interpreted as an indirect measure of engagement.

Finally, we decided to collect verbal expressions for the assessment of communication events between the participants and the robot. In particular, for “conceptual use”, it was intended that all the communication events were related to service execution, while for “conversional interaction”, it was intended all the communication events expressed a willingness to have a friendly talk with the robot, for example. Moreover, all the free statements of the users were collected and coded as acceptability contents when they expressed enjoyment/anxiety during the task and predisposition toward the system, and as HRI indicators when they were related to the robot itself or one of its features.

### 2.2. Equipment

For the testing sessions, the DomoCasa Lab was equipped with two Zig-Bee wireless sensor networks. The elevator was embedded in the Robot-Era system through a Phidget input/output digital board used to control it remotely. More details about Robot-Era architecture are explained in [29,30]. Users interacted with the system through the graphical user interface on the robot’s tablet, or through the speech user interface, with a wearable microphone connected to a speech recognition software module [31].

## 3. Results

The interaction sessions started when the user first called DORO and ended when DORO left, after having received the command. Table 1 reports the mean duration of each service and the mean percentage spent interacting with the robot.

The results of the behavioral analysis revealed a largely positive attitude from the whole sample toward the robot, which was inferred from nonverbal clues and nonverbal emotional expressions. Taking into consideration the nonverbal communication and expressed enjoyment, Communication and Garbage seemed to have been more appreciated by the users, even if the participants seemed to spend more time in interaction during Shopping (Table 2).

Even if the video analysis showed the absence of relevant problems related to lack of acceptability, the presence of the tablet as a principal mean of interaction may have reduced opportunities for communicating or just looking at the robot, as opposed to the Communication and Garbage services, which required less use of the tablet, leaving the participants free to communicate. Despite this, it should be said that only one participant experienced anxiety during the performance of Shopping task, mostly shown by emotional reaction during the execution—gaze directed to the facilitator for support—while twelve older adults mainly focused on the robot through gaze and body orientation. In one case, one user started walking around DORO. Finally, one user touched DORO’s fingers, looking for physical interaction as shown by her statements (“I’d like the robot to have a human-like arm, to be adapted to my apartment”; “Does he talk if I touch him?”).

From the analysis of the communication events during Shopping, the participants that had a higher degree of familiarity with technology (nine out of thirty-five) showed a positive predisposition towards the tablet, and provided an interesting corpus of statements for updating the tablet graphic interface. Moreover, sixteen participants expressed enjoyment in using DORO, and appreciation for its physical appearance (“From today on, I think I prefer the robot, because the tablet is still a little complicated for me. I need to exercise more”; “I really like the robot with this face!”). From the analysis of the communication events, the statements showed a high degree of human–robot interaction (i.e., by laughing and joking with DORO: “Thanks for coming here DORO!”; “You are arrived very soon ORO, thank you!”; “Let’s take a picture with DORO.”). On the acceptability, the statements collected revealed an overall positive evaluation of the tasks (“It could be useful if DORO would bring the shopping bags inside the home”; “I do not think that the shopping service is too slow. If I was at home, I could do other things while he shops for me”). Only two participants reported a negative evaluation of the system. From an analysis of the communication contents of HRI, the majority of the users seemed to like DORO and its features (“I think DORO’s speed is appropriate for the domestic environment”; “I like DORO’s voice because it is not too metallic”) and looked for more conversational communication (“Is it possible to use courtesy communication?”, “I think that it would be better if the robot had vocal commands, because if I have to stay in bed, it is difficult to get the tablet”, “I think I prefer the robot, because tablet is still a little complicated for me. I need to exercise more”).

Regarding the Communication service, we observed that the participants were more engaged in this task as opposed to the others, due to the higher percentage of directed gaze and open posture (see Table 1). Of the thirty-five subjects, thirteen showed enjoyment during the task from the analysis of facial expressions and gestures. In addition, it can be said that the opportunity to receive warnings in time was considered to be the favorite functionality of the entire system by almost all the subjects (“I think it is useful to receive warnings on that—gas leak”; “DORO should go faster, if there is a gas leak!”).

From the analysis of verbal interaction, only five persons reported not feeling confident with this service before starting the test. One of the most common difficulties encountered by the participants during communication was how to deal with the warnings (specifically observed with three users): after a warning was given, they did not know what to say/do with DORO. This problem was interpreted as a bias caused by the double tasks required by the use case—to understand the warning received and to answer the incoming call—or by the lack of control over the event/process: the user did not receive any feedback on how the robot was going to act (calling someone), for example. On acceptability, the users expressed a wish of having more complex functionalities associated with the warnings (“Can the robot help in case of intruders in the home?”). Looking at the percentage of nonverbal emotional behaviors, the garbage collection was perceived as the funniest one: the video analysis shows that twenty-two participants enjoyed the cooperation with the robot and spent time in friendly communication with DORO. Within the most relevant statements, a high rate of acceptability was deduced (“I think that this service is very useful!”; “This service is interesting”), as well as a willingness to have more features, in order to perform complex tasks (“Can the robot differentiate the garbage?”). Finally, one user requested more support, to transport heavy bags. Relevant statements were also collected about DORO’s features (“The robot does not seem more only a machine”; “DORO has a really nice head!”) and, in one case, the robot also evoked an emotional reaction in the user (“I felt tenderness for him”).

## 4. Discussion

Due to the experimental protocol, interaction with the robot was mostly conceptual: the users were asked to accomplish some specific tasks, and because of this, their use of the system was subjected to some constraints. Nevertheless, the duration of certain tasks was left exclusively to the users, who, in some cases, demonstrated interest in the opportunity of talking more with the robot, posed questions directly to DORO, or expressed willingness to spend some time with it. Even though the participants mostly seemed quiet while interacting, their emotional status was never negative, and they sometimes enjoyed the interaction. It is important to stress that the robot is not supposed to represent a social companion, and, consequently, it is not supposed to arouse amusement and enjoyment. Even if the communication with the robot was formal, many users demonstrated good comprehension overall and good management of the command to be given, as well as friendly communication.

In general, a connection between expressed enjoyment and the production of verbal statements on the robot’s appearance was observed, which may suggest a role of some personality traits in supporting the acceptability of the platform. Finally, what emerged was an overall positive attitude toward the robot interaction, and a good degree of trust and engagement that eventually seemed to not be affected by environmental or methodological issues.

Despite having defined the interaction as neutral, it is possible to stress the importance of some psychosocial determinants that can play a crucial role in positive robot acceptance. In our case, we observed that the perceived usefulness of the services offered by the Robot-Era platform, the attitude toward technology due to successful past experience, and the presence of some personality traits should represent a starting point for designing strategies to support older-adult–robot interaction. Nevertheless, the study presents relevant limitations in relation to the collection of factors that can determine acceptability of technology, such as digital literacy, educational level, and other socio-demographic characteristics that need to be deeply investigated.

As lessons learnt, we suggest that industries spend resources in developing robotic solutions able to accomplish a few specific and simple tasks, assuring a high degree of usability, as the perceived usefulness, in particular, may generate too-high expectations in end-users, leading to frustration and misuse if the system fails.

On the end-users’ side, one strategy to promote acceptance and to modulate the perceived usefulness would be the provision of training dedicated to technological literacy. Starting from the initial representations of technology, adequate technology literacy training should provide a comprehensive understanding on how technology and robotics can improve daily life, and when it cannot [10,14,22,32,33].

## 5. Conclusions

The purpose of this pilot was to suggest strategies for the promotion of older adult–robot interaction, starting with an analysis of the older users’ behaviors toward the Robot-Era platform. The analysis of the results summarized important aspects to be taken into account by industries and research, for example, the need for providing robotic solutions that respect the tasks they offer to users, by giving assistance from the beginning to the end of the service flow. In addition, it is necessary to give older consumers dedicated training in technological literacy that can guarantee proper, long-lasting, and successful use.

## Figures and Tables

**Table 1:** Interaction percentage during the test sessions.

Service	Duration of the Test Session	Percentage of the Interaction
Communication	4 min (±1.15)	69%
Shopping	5 min 20 s (±1.67)	81%
Garbage	3 min (±2.18)	67%

**Table 2:** Mean percentages of time for each coded behavior.

**Communication**	Nonverbal behaviors	Gaze	Directed	89.5%
			None	10.5%
		Proxemics	Intimate/personal	64%
			Public	0%
			Default posture	36%
	Nonverbal emotional behaviors	Emotion	Enjoyment	12%
			Anxiety	0%
			Neutral	88%
	Verbal expressions	Communication	Conceptual use	98.2%
			Conversional	1.8%
**Shopping**	Nonverbal behaviors	Gaze	Directed	75%
			None	25%
		Proxemics	Intimate/personal	12%
			Public	0%
			Default posture	88%
	Nonverbal emotional behaviors	Emotion	Enjoyment	4%
			Anxiety	1%
			Neutral	96%
	Verbal interaction	Communication	Conceptual use	99%
			Conversional	1%
**Garbage**	Nonverbal behaviors	Gaze	Directed	74%
			None	26%
		Proxemics	Intimate/personal	0%
			Public	12%
			Default posture	88%
	Nonverbal emotional behaviors	Emotion	Enjoyment	30%
			Anxiety	0%
			Neutral	70%
	Verbal interaction	Communication	Conceptual use	96%
			Conversational	4%

## Data Availability

The datasets generated, used, and analyzed during the trial and its preceding pilot trial are or will be available from the corresponding author upon reasonable request.
